# Risk of Excess and Inadequate Gestational Weight Gain among Hispanic Women: Effects of Immigration Generational Status

**DOI:** 10.3390/ijerph17186452

**Published:** 2020-09-04

**Authors:** Sajeevika S. Daundasekara, Daniel P. O’Connor, Jodi Berger Cardoso, Tracey Ledoux, Daphne C. Hernandez

**Affiliations:** 1 Department of Health & Human Performance, The University of Houston, Houston, TX 77204, USA; ssdaunda@Central.UH.EDU (S.S.D.); dpoconno@central.uh.edu (D.P.O.); taledoux@Central.uh.edu (T.L.); 2 HEALTH Research Institute, The University of Houston, Houston, TX 77204, USA; 3 Graduate College of Social Work, The University of Houston, Houston, TX 77204, USA; jcardoso@central.uh.edu; 4 Department of Research, Cizik School of Nursing, University of Texas Health Science Center, Houston, TX 77030, USA

**Keywords:** first-generation, second-generation, third-/higher-generation, immigrant, NLSY79, pregnancy, gestational weight gain

## Abstract

There is a dearth of information on the risk of inadequate and excess gestational weight gain (GWG) among different generations of Hispanic women in the United States. Therefore, the objective of this study was to understand the relationship of GWG and immigration across three generations of Hispanic women. The study was conducted using data from National Longitudinal Survey of Youth 1979 (NLSY79). The study sample included 580 (unweighted count) women (148 first-generation, 117 second-generation, and 315 third-/higher-generation). Sociodemographic and immigration data were extracted from the main NLSY79 survey, and pregnancy data were extracted from the child/young adult survey following the biological children born to women in NLSY79. Covariate adjusted weighted logistic regression models were conducted to assess the risk of inadequate and excess GWG among the groups. Average total GWG was 14.98 kg, 23% had inadequate GWG, and 50% had excess GWG. After controlling for the covariates, there was no difference in the risk of inadequate GWG between the three generations. First-generation women (OR = 0.47, *p* = 0.039) and third-/higher-generation women (OR = 0.39, *p* = 0.004) had significantly lower risk of excess GWG compared to second-generation women. It is important to recognize the generational status of Hispanic women as a risk factor for excess GWG.

## 1. Introduction

Overweight and obesity prevalence rates are high among childbearing women [1,2], especially among Hispanic women [3]. Further, research evidence has shown that, among Hispanic women, the rate of inadequate gestational weight gain (GWG) is 17–30% and the rate of excess GWG is 36–52% and that this varies by maternal origin [4,5,6,7,8]. Entering pregnancy with excess weight places women and their unborn baby at risk for health complications. Further, gaining inadequate or excess weight during pregnancy also increases the health risks to both the mother and the child [9,10]. This includes gestational diabetes, hypertension, preterm deliveries, cesarean delivery, fetal growth restrictions, fetal macrosomia, large for gestational age babies, small for gestational age babies, neonatal hypoglycemia, and infant and childhood obesity [11,12,13]. Consequently, gaining excess weight during pregnancy is related to postpartum weight retention and, later, is related to obesity prevalence rates [14,15]. To optimize maternal and child health outcomes, the Institute of Medicine (IOM) revised the GWG recommendations in 2009. According to the latest guidelines, women with pre-pregnancy underweight, normal weight, overweight, and obese weight status should gain 28–40 lbs., 25–35 lbs., 15–25 lbs., and 11–20 lbs., respectively, for singleton pregnancies [16].

A number of studies have shown that pregnancy outcomes differ between immigrant and U.S.-born women even after controlling for potential confounding variables [17,18]. In general, women who immigrate to the U.S. have higher rates of maternal mortality, fetal growth restrictions, infections, and poor use of prenatal care [19,20]. On the other hand, first-generation Hispanic women have also shown similar or better rates of preterm birth and low birth weight compared to U.S.-born women [21,22,23]. This is referred to as an epidemiological paradox [22]. These results could considerably vary due to the immigrants’ country of origin and to social and cultural factors. Protective social and cultural factors such as higher family cohesion and values, higher social support, better maternal diets, and lower levels of smoking during pregnancy may explain positive pregnancy outcomes among Hispanic immigrants [24,25,26,27].

However, there are limited studies comparing GWG and the risk of inadequate/excess GWG among Hispanic immigrant women across generation status. Instead, the research has focused on the risk of excess GWG among Hispanic women based on their *nativity* and the findings are inconsistent. Sangi-Haghpeykar et al. [28] reported that U.S.-born Hispanic women had greater risk of excess GWG compared to foreign-born Hispanic women. In contrast, Siega-Riz and Hobel [5] reported that being U.S. born decreased the risk of poor total GWG among Hispanic women.

To our knowledge, there are no studies evaluating GWG among different generations of Hispanic women or identifying generations that are at risk of inadequate GWG and excess GWG using a nationally representative sample of Hispanic women of different ethnic origins. To develop effective methods for controlling weight gain during pregnancy among Hispanic women, it is important to understand how much weight gain occurs during pregnancy by immigrant generation status. Therefore, the first aim of the study was to assess whether there are significant differences in total GWG and GWG adequacy among the three Hispanic immigration groups (first-generation, second-generation, and third-/higher-generation). Further, it is important to investigate whether immigrant generation status is a protector/risk factor against inadequate and excessive GWG independent of their sociodemographic and pregnancy characteristics. Thus, the second aim of the study was to evaluate whether the risk of inadequate and excess GWG is different among the three generations of Hispanic women after controlling for potential confounding variables. Even though the findings among Hispanic women are inconsistent, based on previous literature that suggest first-generation immigrants in general are more likely to abide by weight gain guidelines [29] and have better pregnancy outcomes than later generation women [21,22,23], we hypothesized that first-generation Hispanic women have lower risk of inadequate and excess GWG compared to second-generation and third-/higher-generation Hispanic women. While prior research has not focused on GWG of second-generation immigrants, assimilation theory does provide guidance in predicting GWG among second-generation Hispanic immigrants. Assimilation theory suggests that the initial differences between immigrants and U.S.-born generations disappear with time and across generations [30,31,32]. Thus, it is hypothesized that third-/higher-generation Hispanic women have similar risk of inadequate and excess GWG compared to second-generation Hispanic.

## 2. Materials and Methods

### 2.1. Study Sample

Data from the National Longitudinal Survey of Youth 1979 (NLSY79) and NLSY79 child and young adult were used in this study. The NLSY79 cohort includes a sample of 12,686 men and women who were born between 1957 and 1964. The cohort was oversampled for Hispanics, blacks and economically disadvantaged nonblack and non-Hispanics. The data were collected through questionnaire-guided interviews annually through 1994 and then biannually. In 1986, a new survey of all children born to women in the NLSY79 cohort began and collected pregnancy-related data and child-specific information. Participants were provided with confidentiality and consent information, and verbal consent was obtained at the beginning of interviews [33]. The institutional review boards at the institutions that manage and conduct the surveys (Ohio State University and the National Opinion Research Center (NORC) at the University of Chicago) have reviewed and approved the surveys [33]. The data is deidentified and publicly available. Therefore, institutional review board approval was not required for the current study.

**Figure 1:**
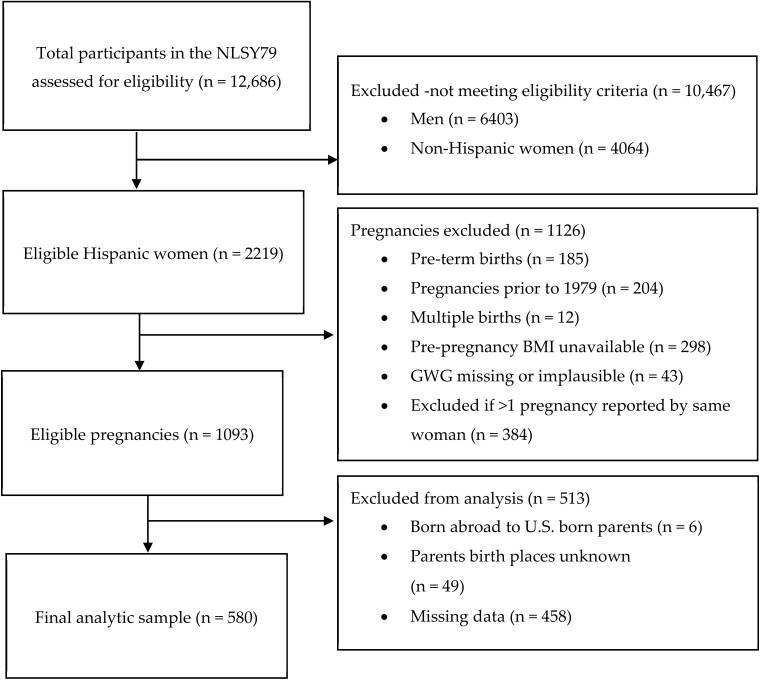
The study sample selection process (the unweighted counts are reported).

For the purpose of the current study, NLSY79 child survey data were combined with their mother’s data. The current study sample included pregnancies after 1979 reported by Hispanic women. The eligible sample included women with at least one singleton, term birth with complete information to calculate pre-pregnancy body mass index (BMI) and GWG. Women with multiple pregnancies (twins/triplets), preterm deliveries (delivered <37 weeks gestational age), women without complete information to calculate pre-pregnancy BMI or GWG, women born abroad to U.S. born parents, and those who did not report parents’ birthplace were excluded from this study (Figure 1). Further, women with missing data for the study variables were also excluded from the current analytic sample. If women reported more than one pregnancy, only the first pregnancy meeting the eligible criteria was used in the current analysis. The analytic sample consisted of an unweighted count of 580 Hispanic women.

### 2.2. Measures

#### 2.2.1. Gestational Weight Gain (GWG)

Women self-reported their weight just before delivery (question in the survey: “What was your weight just before you delivered?”) in every survey beginning in the year 1986. Total GWG was calculated by subtracting the recalled pre-pregnancy weight from the reported weight just before delivery [34]. Women with negative GWG values were removed from the final data set as there were no medical record data to confirm weight loss during pregnancy. GWG adequacy was defined as the ratio of observed GWG to expected GWG at gestational age (reported in weeks by mother) at delivery [35,36,37]. The GWG adequacy and the expected GWG was calculated using the following equations:

$$\mathrm{GWG adequacy}=\left(\mathrm{observed GWG}/\mathrm{expected GWG}\right)\times 100$$

$$\mathrm{Expected GWG}=\mathrm{recommended first-trimester total weight gain}+\left(\mathrm{gestational age at delivery}-13\right)\times \mathrm{recommended rate of gain in second and third trimesters}$$

Women were categorized by the percentage of GWG recommendations met as inadequate, adequate, or excess based on the IOM 2009-recommended GWG range for their pre-pregnancy weight status as described previously [35,36,37]. As an example, the range of recommended GWG at term for normal-weight women is 11.5–16 kg, and this corresponds to 86–120% of the IOM recommendation. Normal-weight women who gained <86% weight of the recommended is considered inadequate GWG, and those who gained >120% weight of the recommendation is considered excess GWG. Two dichotomous variables were created to evaluate the risk of inadequate and excess GWG, using adequate GWG category (within the 86–120% range) as the reference group.

#### 2.2.2. Immigration Generational Status

Women were categorized as first-generation, second-generation, and third-/higher-generation based on their place of birth and their parents’ birthplace. Women born outside the U.S. were coded as first-generation immigrants; women born in the U.S. with at least one biological parent born outside the U.S. were coded as second-generation immigrants. Women born in the U.S. with both parents born in the U.S. were coded as third-/higher-generation.

#### 2.2.3. Covariates

The following sociodemographic and pregnancy characteristics available in the NLSY79 were used as covariates in the analysis. These variables were selected a priori based on the literature indicating associations with GWG and immigration generational status [4,5,15,29,36]. Sociodemographic characteristics including maternal age at child’s birth (18 years or less (reference category), 19–29 years, or 30 years or more) and ethnic origin (Puerto Rican, Mexican, or other (reference category)) data extracted from the initial survey in 1979. Information on marital status (married (reference category) vs. other), education (less than high school, high school, college, or higher (reference category)), employment status (employed (reference category) vs. unemployed/out of labor force), and poverty status (in poverty vs. not in poverty (reference category)) was collected from the survey of the year closest to the time of pregnancy. Pregnancy characteristics included child birth order, pregnancy alcohol use (never drink (reference category), drink during pregnancy, and quit during pregnancy), pregnancy smoking (never smoked (reference category), smoked during pregnancy, and quit during pregnancy], month of first prenatal care visit (first trimester vs. later (reference category)), and pre-pregnancy weight categories. The pre-pregnancy weight categories were created based on the Center for Disease Control and Prevention (CDC) classification using the BMI calculated from self-reported pre-pregnancy weight and height (<18.5 underweight, 18.5–24.9 normal weight (reference category), 25–29.9 overweight, and ≥30 obese). The models were also controlled for the interview language (English (reference category) vs. Spanish), which is a proxy measure for acculturation [38].

### 2.3. Statistical Analysis

Bivariate comparisons were conducted between immigration groups for total GWG, GWG adequacy, and covariates using Kruskal–Wallis H test (for continuous variables that are not normally distributed) and chi-square tests (for categorical variables). To evaluate whether the three immigration generations have significantly different risks of inadequate and excess GWG, two weighted univariate logistic regression analyses were performed to control for maternal age, ethnic origin, marital status, education, employment, poverty status, birth order, pregnancy alcohol consumption, pregnancy smoking, prenatal care, pre-pregnancy weight category, and interview language identified a priori as potential covariates. Prior to conducting the analysis, the assumptions of logistic regression were evaluated and confirmed. All analyses were performed with Stata version 15.

## 3. Results

The final analytic sample for the current study consists of 580 (unweighted count) Hispanic women reporting a pregnancy during 1979–2014. There are 148 (24.4%) first-generation, 117 (19.7%) second-generation, and 315 (56.0%) third-/higher-generation Hispanic women. The sample characteristics are shown in Table 1. The majority of pregnancies were reported by married (68%) women, with high school (40%) education during 1980–1989. The mean total GWG of the sample was 14.98 kg (standard error (SE) = 0.30 kg) with 50% gaining excess weight and 23% gaining inadequate weight. According to the Kruskal–Wallis H test, there was a significant difference in the total GWG between first-generation Hispanic women and second-generation women (χ2(1) = 5.929, *p* = 0.015). Among first-generation immigrants, 43% had excess GWG, 30% gained weight adequately, and 26% gained weight inadequately. Similarly, second-generation immigrants’ rates of excess, adequate, and inadequate GWG were 60%, 20%, and 20%, respectively. Forty-nine percent of third-/higher-generation women had excess GWG, while 30% gained adequately and 22% gained inadequately. Second-generation women had significantly higher rates of excess GWG compared to first generation (χ2 = 6.18, *p* = 0.013).

### 3.1. Immigration Generation Predicting Inadequate GWG Among Hispanic Women

According to the weighted logistic regression model, after controlling for covariates, none of the immigration generational statuses were significant predictors of risk of inadequate GWG (Table 2). The follow-up tests showed that the odds ratio for inadequate GWG was not significantly different between first-generation and third-/higher-generation Hispanic women (F = 0.44, *p* = 0.507). Only maternal age, Puerto Rican origin, and pre-pregnancy overweight weight status were significant unique predictors of inadequate GWG among Hispanic women. Women aged 30 years or higher had lower risk of inadequate GWG (OR = 0.39, *p* = 0.042) compared to women age 19–29 years. Overweight women were less likely to gain inadequate weight compared to normal weight women (OR = 0.32, *p* = 0.008), and compared to women of other Hispanic/Latino origins, women of Puerto Rican origin had higher risk of inadequate GWG (OR = 3.72, *p* = 0.029).

### 3.2. Immigration Generation Predicting Excess GWG Among Hispanic Women

According to the weighted logistic regression model predicting risk of excess GWG, after controlling for covariates, first-generation (OR = 0.47, *p* = 0.039) and third-/higher-generation (OR = 0.39, *p* = 0.004) Hispanic women had significantly lower odds of excess GWG compared to second-generation (Table 2). The follow-up tests showed that the odds ratio for excess GWG was not significantly different between first-generation compared to third-/higher-generation Hispanic women (F = 0.30, *p* = 0.584). Further, maternal origin, marital status, poverty, and pre-pregnancy weight status were significant unique predictors of excess GWG. Hispanic women with a Puerto Rican origin were significantly more likely to gain excess GWG compared to women with other Hispanic/Latino origins (OR = 2.79, *p* = 0.027). Single women were more likely to gain excess GWG compared to married women (OR = 2.80, *p* < 0.001). Women in poverty had significantly higher odds of excess GWG compared to those not in poverty (OR = 56, *p* = 0.046). Pre-pregnancy overweight women (OR = 1.47, *p* = 0.022) and obese women (OR = 6.65, *p* = 0.014) were more likely to gain excess GWG compared to normal weight women.

## 4. Discussion

The current study was designed to assess differences in total GWG and GWG adequacy among three generations of Hispanic immigrants (first-generation, second-generation, and third-/higher-generation) and to evaluate the risk of inadequate and excess GWG among three generations of Hispanic women after controlling for the potential confounding variables. Preliminary bivariate analysis showed that first-generation Hispanic women gained lower total weight and had lower rates of excess GWG compared to second-generation Hispanic women. In terms of the likelihood of inadequate GWG, the regression model showed no significant differences between first-generation and second-generation women or between first-generation and third-/higher-generation Hispanic women. The risk of inadequate GWG was similar between second-generation women and third-/higher-generation women. Similar to the current findings, Sangi-Haghpeykar, Lam, and Raine [28] reported that, among Hispanic women, there was no significant differences in the rate of inadequate GWG based on the nativity of Hispanic women. However, the risk of excess GWG varied significantly by immigration generation status of Hispanic women. First-generation Hispanic women had lower risk of excess GWG compared to second-generation women. Opposed to our hypothesis, the risk of excess GWG was not different between first-generation and third-/higher-generation Hispanic women and the risk of excess GWG was lower among third-/higher-generation compared to second-generation women.

The observed higher risk of excess GWG among second-generation Hispanic women compared to first-generation and third-/higher-generation women might be explained by the behavioral and socioeconomic characteristics of the three immigrant generations included in the current study. In general, studies have shown that the second-generation Hispanics (especially of Mexican origin) have poorer health outcomes compared to their first-generation counterparts [24,39]. Compared to U.S.-born counterparts (i.e., second-generation and onwards), first-generation immigrants have healthier dietary patterns, consuming more rice, fruits, and vegetables [40,41]. First-generation immigrants are more likely to live in areas with higher immigrant density and with reported greater linguistic isolation, which could slow the acculturation process protecting native ethnic group values and norms. In the current study, first-generation Hispanic women were less acculturated (>25% of the interviews among first-generation women were conducted in Spanish, which is a proxy for acculturation), while second-generation Hispanic women reported higher acculturation (all interviews were conducted in English); thus, this behavior could explain why second-generation Hispanic women were at higher risk for excess weight gain.

Research focused on the socioeconomics has suggested that having college-level or higher education is associated with higher risk of excess GWG among Hispanic women [42]. Further, according to Chu et al. [43], women with <12 years of education were less likely to gain excess weight during pregnancy compared to women with more education. Min et al. [44] reported that both high education and having a routine job were associated with excessive GWG. In the current study, first-and third-/higher-generation Hispanic women had lower education and lower rates of employment compared to second-generation Hispanic women. Thus, the lower socioeconomic status among first- and third-/higher-generation Hispanic women in the current study might explain their lower risk of excess GWG in comparison to second-generation women.

Studies among second-generation immigrants have reported that they are more acculturated to the host culture and have reported unhealthy behaviors, such as smoking [39,45]. Studies have shown that women who continued to smoke during pregnancy gained lower GWG compared to women who quit smoking during pregnancy [46,47]. In the current study, over 20% of third-/higher-generation Hispanic women engaged in smoking during pregnancy. Engaging in unhealthy behaviors such as smoking in combination with having lower socioeconomic status might explain the lower risk of excess GWG among third-/higher-generation Hispanics women compared to second-generation women.

### 4.1. Limitations and Strengths

In the existing sample, the majority of the first-generation immigrants had been in the U.S. for >10 years by the time of first pregnancy (results not shown). Therefore, rather than focusing solely on generation status, future research should account for length of residency (i.e., migration to the U.S. as an adolescent/adult (≥13 years of age) versus migration to the U.S. as a child/adolescent (≤12 years of age)) and visa/residency status to account for the variation in first-generation immigrants. This may provide an in-depth understanding of GWG among first-generation immigrants. Last, distal measures, such as policy and environmental differences, which include but are not limited to immigrant integration policies, health policies, and obesogenic environment, along with proximal measures, such as perceived stress and social support, were not available in the data. These measurements might be potential explanations for inadequate and excess GWG among immigrants in the U.S.

Aside from the limitations stated above, the majority of pregnancies in the current study were reported before 1990 and those women were not under auspiciousness of the 2009 GWG recommendations. The 2009 guidelines established upper limit GWG recommendations because it was discovered that pregnancy risks increase above that threshold regardless of when pregnancies occurred. Nevertheless, the 2009 GWG recommendations improve the validity of the results as it gives an upper limit for GWG for all pre-pregnancy BMI categories. In addition, all height and weight measurements were self-reported, which could introduce bias. However, Ranchod, Headen, Petito, Deardorff, Rehkopf, and Abrams [34] assessed the reliability of self-reported weight data in the NLSY79 by comparing the pre-pregnancy weight data with the weight reported in the survey 1–2 years prior to pregnancy and reported reasonable agreement. Further, numerous previous researchers have used the available self-reported height and weight data in the NLSY79 as a valid and reliable measure when measured variables are unavailable [48,49].

One of the major strengths of this study is the use of a nationally representative longitudinal dataset with adequate sample sizes of the three generations of immigrants that has been used in many studies [34,50,51,52]. The original NLSY79 sample (unweighted count with data to determine immigration status = 12,348) consists of 789 first-generation immigrants (weighted percent = 3.98%), 797 second-generation immigrants (weighted percent = 5.40%), and 10,764 third-/higher-generation immigrants or nonimmigrants (weighted percent = 90.62%). The mode of data collection for this data set was a questionnaire-guided interview. This reduces the errors in reporting as the interviewer could clarify questions for the respondents and reduces missing data. The respondents were also able to select the preferred language of interview, which also improves the validity of responses.

### 4.2. Implications for Practice and Policy

Currently, there is limited evidence on the risk of unhealthy GWG among Hispanic immigrant women of first, second, and third/higher generation in the United States. This information is important in order to identify at-risk women as early as possible in their pregnancy and to provide them with the required support to ensure a safe and healthy pregnancy. Our results show that immigration generational status was not associated with risk of inadequate GWG and that second-generation Hispanic women are at risk of excess GWG compared to other two groups. Therefore, second-generation Hispanic women may require guidance on healthy weight gain during pregnancy and should be provided with the opportunity to participate in GWG control interventions.

Most of the identified risk factors for inadequate and excess GWG are non-modifiable after pregnancy begins. Thus, more sustained approaches to improving women’s health across the lifespan could be more beneficial to controlling for inadequate and excess GWG compared to interventions provided during pregnancy. A stage of developmental transition, such as pregnancy, is considered a “teachable moment” for women as they are motivated to spontaneously adopt risk-reducing health behaviors [53]. Adolescence is also another stage of developmental transition and could be considered a “teachable moment” as it is the stage in which pregnancy becomes feasible. While not all adolescents are going to become pregnant, adolescence compared to adulthood does provide an earlier opportunity to motivate young women to make lifestyle changes regarding nutrition and physical activity to maintain weight and to prevent weight gain. Intervening during adolescence might have intergenerational benefits, which could lead to reduced obesity among future generations [54].

## 5. Conclusions

Nearly 50% of Hispanic women in the sample had excess GWG, which is a substantial percentage of women. These women and their children are at risk of future obesity and metabolic disease. After controlling for the effects of age, ethnic origin, marital status, employment, education, poverty, birth order, pre-pregnancy BMI, pregnancy alcohol use, smoking, use of prenatal care, and interview language, first- and third-/higher-generation Hispanic women were at lower risk of excess GWG compared second-generation Hispanic women. Further analyses are required to determine whether socioeconomic status and pregnancy characteristics might explain the current study findings.

## Figures and Tables

**Table 1:** Sociodemographic characteristics and pregnancy characteristics by immigration generation given as weighted percentage or mean (SE).

Characteristic	Total Sample (n = 580)	First-Generation (n = 148)	Second-Generation (n = 117)	Third-/Higher-Generation (n = 315)
Outcome variables				
Total GWG (kg)	14.89 (0.30)	13.82 (0.51) ^a^	16.19 (0.66)	15.08 (0.42)
GWG adequacy				
Inadequate	22.55%	26.17%	20.23%	21.78%
Adequate	27.76%	30.36%	19.73%	29.45%
Excess	49.69%	43.47% ^a^	60.04%	48.77%
Predictor variable				
Immigration generations				
First-generation	24.40%	100.00%	-	-
Second-generation	19.65%	-	100.00%	-
Third-/higher-generation	55.95%	-	-	100.00%
Sociodemographic characteristics				
Maternal age at birth				
18 years or less	6.99%	6.20%	5.21%	7.95%
19–29 years	77.97%	78.06%	82.26%	76.43%
30 years or more	15.04%	15.74%	12.53%	15.62%
Ethnic origin				
Puerto Rican	12.49%	0% ^b^	2.40% ^c^	21.48%
Mexican	55.72%	58.58% ^b^	70.57% ^c^	49.25%
Other	31.79%	41.42% ^a^	27.03%	29.27%
Marital status at birth				
Married	68.26%	74.14%	72.05%	64.37%
Single, divorced, separated, or widowed	31.74%	25.865	27.95%	35.63%
Level of education at birth				
Less than high school	32.63%	45.54% ^a,b^	24.13%	29.99%
High school	39.61%	25.26% ^b^	33.42% ^c^	48.03%
College or higher	27.76%	29.20% ^a^	42.46% ^c^	21.98%
Employment status at birth				
Employed	48.84%	42.57% ^a^	55.75% ^c^	49.14%
Unemployed/out of labor force ^d^	51.16%	57.43%	44.25%	50.86%
Household poverty status at birth ^e^				
In poverty	25.07%	27.10%	23.89%	24.59%
Maternal pre-pregnancy weight status				
Pre-pregnancy weight category				
Underweight	7.27%	5.89%	6.45%	8.32%
Normal	67.45%	70.49%	69.26%	65.49%
Overweight	19.38%	19.59%	16.30%	20.37%
Obese	5.90%	4.04%	7.99%	5.97%
Pregnancy characteristics				
Birth order	1.59 (0.04)	1.66 (0.08)	1.47 (0.08)	1.61 (0.05)
Alcohol consumption				
Never drink	59.69%	74.50% ^a,b^	56.28%	54.43%
Quit during pregnancy	10.06%	8.59%	12.24%	9.94%
Drink during pregnancy	30.25%	16.91% ^a,b^	31.47%	35.63%
Smoking				
Never smoked	80.37%	89.02% ^b^	85.47% ^c^	74.81%
Quit during pregnancy	3.17%	2.21%	4.58%	3.10%
Smoked during pregnancy	16.46%	8.77% ^b^	9.94% ^c^	22.10%
First prenatal care visit				
In first trimester	76.48%	74.74%	83.22%	74.87%
Second trimester or later	23.52%	25.26%	16.78%	25.13%
Gestational age at delivery (weeks)	39.30 (0.06)	39.11 (0.09)	39.13 (0.11)	39.44 (0.10)
Interview characteristics				
Interview language				
English	92.44%	72.34% ^a,b^	100.00%	98.55%
Spanish	7.56%	27.66%	0.00%	1.45%

Bivariate comparisons were conducted using Kruskal–Wallis H test for continuous variables that are not normally distributed and chi-square tests for categorical variables. ^a^ First-generation is different from second-generation, *p* < 0.05 ^b^. First-generation is different from third-/higher-generation, *p* < 0.05. ^c^ Second-generation is different from third-/higher-generation, *p* < 0.05. ^d^ Out of labor force: own-home housework, in school, unable to work because of long-term physical or mental illness, and retired ^e^ In the National Longitudinal Survey of Youth 1979 (NLSY79), participants were determined to be in poverty if total net family income for the last calendar year was below the poverty income guidelines given their family size.

**Table 2:** Multivariate logistic regression of immigration generations status predicting the risk of inadequate and excess gestational weight gain (GWG; unweighted count = 580).

Characteristic	Inadequate GWG		Excess GWG	
	Odds Ratio (95% CI)	*p* Value	Odds Ratio (95% CI)	*p* Value
Predictor variable				
Immigration category (ref = Second-generation)				
First-generation	0.82 (0.37, 1.83)	0.632	0.47 (0.23, 0.96)	0.039
Third-/higher-generation	0.65 (0.32, 1.33)	0.242	0.39 (0.21, 0.74)	0.004
Covariates				
Maternal age at child’s birth (ref = 19–29 years)				
18 years or less	1.93 (0.66, 5.61)	0.229	1.04 (0.38, 2.83)	0.934
30 years or more	0.39 (0.16, 0.97)	0.042	0.82 (0.42, 1.62)	0.566
Ethnic origin (ref = Other Hispanic/Latino)				
Puerto Rican	3.72 (1.15, 12.03)	0.029	2.79 (1.12, 6.94)	0.027
Mexican	1.38 (0.77, 2.49)	0.281	0.94 (0.56, 1.58)	0.818
Marital status (ref = married)				
Single, divorced, separated, or widowed	0.86 (0.45, 1.65)	0.654	2.80 (1.59, 4.92)	<0.001
Education (ref = college or higher)				
Less than high school	0.87 (0.37, 2.06)	0.752	1.07 (0.53, 2.19)	0.844
High school	1.00 (0.50, 2.01)	0.996	1.27 (0.69, 2.32)	0.445
Employment status (ref = employed)				
Unemployed/out of labor force ^a^	1.17 (0.62, 2.21)	0.626	1.02 (0.61, 1.71)	0.931
In poverty	0.96 (0.49, 1.87)	0.897	0.56 (0.31, 0.99)	0.046
Pre-pregnancy weight status (ref = normal weight)				
Underweight	0.98 (0.42, 2.28)	0.961	0.58 (0.27, 1.28)	0.179
Overweight	0.32 (0.14, 0.74)	0.008	1.94 (1.10, 3.41)	0.022
Obese	1.04 (0.13, 8.22)	0.973	6.65 (1.48, 29.91)	0.014
Birth order	0.97 (0.67, 1.42)	0.888	1.00 (0.75, 1.33)	0.995
Pregnancy alcohol use (ref = never drink)				
Drink during pregnancy	1.32 (0.67, 2.60)	0.423	1.13 (0.63, 2.02)	0.683
Quit during pregnancy	0.69 (0.26, 1.86)	0.468	0.69 (0.34, 1.40)	0.304
Pregnancy smoking (ref = never smoked)				
Smoked during pregnancy	1.49 (0.68, 3.28)	0.323	1.33 (0.62, 2.89)	0.465
Quit during pregnancy	1.15 (0.17, 7.55)	0.888	1.44 (0.45, 4.59)	0.538
Prenatal care (ref = first visit in second trimester or later)				
First visit in first trimester	0.89 (0.46, 1.71)	0.728	1.45 (0.83, 2.53)	0.192
Interview language (ref = Spanish)				
English	0.42 (0.15, 1.17)	0.097	0.71 (0.24, 2.08)	0.527

^a^ Out of labor force includes own-home housework, in school, unable to work because of long-term physical or mental illness, and retired.
